# Compensatory Serotonin Synthesis and Histone H3 Serotonylation in Preimplantation Embryos Exposed to Maternal Fluoxetine or Monoamine Oxidase Blockade

**DOI:** 10.3390/jdb14020015

**Published:** 2026-04-03

**Authors:** Veronika S. Frolova, Denis A. Nikishin

**Affiliations:** 1 Department of Embryology, Biological Faculty, Lomonosov Moscow State University, 119234 Moscow, Russia; frolova.veronika.2014@post.bio.msu.ru; 2 Koltzov Institute of Developmental Biology RAS, 119334 Moscow, Russia

**Keywords:** serotonin, preimplantation embryo, monoamine oxidase, fluoxetine, serotonylation, epigenetics, reproductive toxicology

## Abstract

Serotonin is a critical morphogen in early development, yet the mechanisms regulating its homeostasis in the preimplantation embryo remain unclear, particularly under conditions of maternal antidepressant exposure. Here, we investigated embryonic serotonergic autonomy using mouse models of pharmacological transport blockade (maternal fluoxetine treatment) and in vitro treatment with the monoamine oxidase inhibitor pargyline. We employed immunofluorescence, RT-qPCR, and live-cell imaging to assess metabolic flux, gene expression, and physiological health. We demonstrate that monoamine oxidase functions as a metabolic firewall, progressively maturing from zygote to blastocyst to degrade excess amines. Paradoxically, maternal serotonin transporter blockade triggered significant intracellular serotonin hyper-accumulation in blastocysts, associated with a trend toward a compensatory upregulation of the biosynthetic gene *Ddc*. While this serotonin overload did not compromise morphology, mitochondrial function, or pluripotency marker expression, it induced a robust epigenetic response. Excess serotonin promoted elevated H3Q5ser immunoreactivity in both nuclear and cytoplasmic compartments via a transglutaminase-dependent mechanism. These findings reveal that the preimplantation embryo possesses a resilient, autonomous serotonergic system capable of compensatory synthesis. However, environmental fluctuations are chemically recorded via transglutaminase-mediated serotonylation, representing an epigenetic mark that warrants further long-term study within the Developmental Origins of Health and Disease (DOHaD) framework.

## 1. Introduction

Serotonin (5-hydroxytryptamine, 5-HT) is a pleiotropic signaling molecule whose biological functions extend far beyond its canonical role as a neurotransmitter in the mature nervous system [1,2,3,4]. In the context of reproduction and embryogenesis, 5-HT acts as a critical morphogen, modulating key developmental transitions well before the formation of neural structures [5,6,7,8,9,10,11,12]. Indeed, the identification of serotonergic system components in early development has led to the consensus that 5-HT orchestrates oocyte maturation, follicle selection, cleavage divisions, and interblastomeric communication [6,13,14,15,16,17,18,19,20]. While the presence of these components is established, the precise mechanisms regulating intracellular serotonin homeostasis—specifically the balance between uptake, synthesis, and degradation—during the preimplantation period remain poorly understood.

Disruptions in serotonin signaling, particularly through pharmacological intervention, provide a powerful lens to study these embryonic mechanisms. Antidepressants, widely prescribed during pregnancy, function by altering neurotransmitter availability via specific transporter blockade or enzymatic inhibition [21]. Selective serotonin reuptake inhibitors (SSRIs), such as fluoxetine, block the serotonin transporter (SERT), thereby preventing 5-HT uptake from the extracellular milieu [22,23,24,25,26]. Conversely, intracellular levels are tightly controlled by monoamine oxidases (MAOs), which catalyze oxidative deamination of monoamines [27,28,29,30]. Among these, Monoamine Oxidase A (MAO-A) is the primary isoform responsible for serotonin degradation [31]. Given that antidepressants exert systemic effects affecting peripheral tissues and the reproductive tract [20,23,32,33,34,35,36,37,38], defining the embryo’s autonomous capacity to handle serotonin fluctuations is critical for understanding reproductive toxicology.

In this study, we investigate the autonomy and plasticity of the preimplantation embryo’s serotonergic system. We utilized pharmacological models to induce states of serotonin overload and deprivation in vitro and in vivo. To simulate intracellular accumulation, we employed pargyline, an irreversible MAO inhibitor previously validated in our model systems [26]. To model altered maternal serotonin distribution, we utilized maternal administration of fluoxetine. Here, we demonstrate that MAO-A is not only present but becomes progressively functional from the zygote to the blastocyst stage, acting as a metabolic gatekeeper. We report that while blocking degradation leads to massive intracellular 5-HT accumulation, this “overload” does not compromise morphology, mitochondrial function, or pluripotency marker expression. Instead, it drives an epigenetic response via transglutaminase-mediated histone H3 serotonylation (H3Q5ser). Furthermore, we uncover a compensatory mechanism wherein maternal fluoxetine exposure triggers premature endogenous serotonin synthesis in the embryo. Collectively, these findings reveal a robust, self-regulating serotonergic machinery in the preimplantation embryo capable of buffering extreme metabolic challenges. While the capacity for endogenous synthesis guarantees preimplantation survival, the resulting epigenetic alterations pose questions for long-term health, highlighting the complex reproductive consequences of pharmacological interventions.

## 2. Materials and Methods

### 2.1. Animals and Ethical Statement

Mature ICR mice (females: 8–12 weeks old, 25–30 g; males: 10–40 weeks old) were obtained from the animal facility of the Koltzov Institute of Developmental Biology RAS. Female mice were randomly assigned to experimental or control groups using a random number generator to minimize selection bias. Animals were housed under controlled environmental conditions (22–24 °C, 14 h light/10 h dark photoperiod) with ad libitum access to food and water. All experimental procedures were conducted in strict accordance with the Council Directive of the European Communities (86/609/EEC) and were approved by the Bioethics Committee of the N.K. Koltzov Institute of Developmental Biology RAS (Protocol No. 68, dated 23 March 2023).

### 2.2. Experimental Design: In Vivo Fluoxetine Treatment

To induce alterations in systemic maternal serotonin distribution, female mice were administered fluoxetine hydrochloride (F844356, Macklin Inc., Shanghai, China) dissolved in drinking water at a concentration of 0.13 mg/mL (equivalent to an estimated dose of 20 mg/kg/day), corresponding to a therapeutic dose range as previously described [20,39]. The pharmacological efficacy and systemic target engagement of this specific in vivo fluoxetine administration protocol have been previously validated by our group; as confirmed by high-performance liquid chromatography (HPLC), this regimen induces robust physiological SERT blockade, resulting in significant alterations in circulating blood serotonin levels [20,39]. However, it must be noted that because maternal blood or local embryonic drug and metabolite concentrations were not directly analytically quantified in the current study cohort, the precise embryonic exposure levels remain theoretical. Treatment was maintained for 10 days prior to mating and continued throughout the preimplantation period. Blastocysts were flushed from the oviducts at 3.5 dpc and processed for downstream analysis. To ensure sample homogeneity, only embryos exhibiting normal morphology (expanded blastocysts with distinct ICM/TE and no signs of fragmentation/degeneration) were included in the analysis. Embryos arrested at earlier stages or showing gross morphological defects were excluded.

### 2.3. Experimental Design: In Vitro Embryo Culture and Treatments

Females were mated with males overnight, and the presence of a vaginal plug was designated as embryonic day 0.5 (0.5 dpc), at which point zygotes were collected. For pharmacological interventions, embryos were cultured in 4-well plates containing KSOM medium until reaching the 2-cell (1.5 dpc), morula (2.5 dpc), or blastocyst (3.5 dpc) stages. To induce intracellular serotonin accumulation, the medium was supplemented with 1 μM 5-HT-creatinine sulfate (H7752, Merck KGaA, Darmstadt, Germany) and 5 μM pargyline hydrochloride (P8013, Merck KGaA, Darmstadt, Germany). To assess endogenous synthesis capacity, embryos were incubated with the precursor 10 μM 5-hydroxy-L-tryptophan (5-HTP, 107751, Merck KGaA, Darmstadt, Germany) in the presence of 5 μM pargyline. The role of transglutaminases was evaluated by adding the inhibitor cystamine dihydrochloride (C121509, Merck KGaA, Darmstadt, Germany) at a concentration of 10 μM. Control groups were maintained in standard KSOM medium. For the in vitro implantation (outgrowth) assay, zygotes were first cultured to the blastocyst stage (4 days) and subsequently transferred to a specialized cellular spread medium [40] where they were cultured for an additional 4 days, corresponding to a developmental age of 8.5 dpc. Outgrowth area was quantified using FIJI/ImageJ 2.9.0/1.54f, open source, available at https://imagej.net/software/fiji/ (URL accessed on 9 March 2026).

### 2.4. RNA Extraction and RT-qPCR

Total RNA was extracted from pools of embryos (4–7 independent pools per condition, with each pool containing 6–10 embryos derived from 3–4 distinct litters) using the guanidine isothiocyanate method (Evrogen, Moscow, Russia) followed by DNase I treatment (Thermo Fisher Scientific, Waltham, MA, USA). cDNA synthesis was performed using random hexamers and MMLV reverse transcriptase (Magnus, Evrogen, Moscow, Russia). Quantitative PCR was carried out on a StepOnePlus System (Thermo Fisher Scientific, Waltham, MA, USA) using qPCRmix-HS SYBR + HighROX (Evrogen, Moscow, Russia). Relative gene expression was calculated using the 2^−ΔΔCt^ method, normalized to the geometric mean of reference genes *Rps18* and *Tbp*. Primer sequences are listed in Table 1.

### 2.5. Immunofluorescence and Confocal Microscopy

Embryos were fixed in 4% paraformaldehyde (PFA) in PBS for 1 h at room temperature or overnight at 4 °C. Following fixation, embryos were washed in PBST (PBS + 0.1% Tween-20). Removal of the zona pellucida and permeabilization were performed using a 1% sodium dodecyl sulfate (SDS) solution. Samples were blocked for 1 h in a buffer containing 3% BSA, 1% fetal calf serum, 0.1% Triton X-100, and 0.01% Tween-20 in PBS. Primary antibodies were incubated overnight at 4 °C: rabbit anti-5-HT (1:1000, S5545 Sigma-Aldrich, St. Louis, MO, USA), rabbit anti-MAO-A (1:1000, ab126751, Abcam, Cambridge, UK), and rabbit anti-H3Q5ser (1:200, A20210, ABclonal, Wuhan, China). Secondary antibodies included FITC-conjugated goat anti-rabbit IgG (1:200, Jackson Immuno Research, West Grove, PA, USA) or goat anti-rabbit IgG-555 (1:300, ABclonal, Wuhan, China). DNA and microfilaments were counterstained with DAPI (1 μg/mL; Merck KGaA, Darmstadt, Germany) and CytoPainter Phalloidin-iFluor 488 (1:1000, Abcam, Cambridge, UK), respectively, for 20 min and then washed four times in PBS. Samples were mounted in Mowiol.

### 2.6. Live-Cell Imaging: Mitochondrial Activity and ROS

For the assessment of physiological status, live blastocysts were incubated with fluorescent probes for 30 min at 37 °C: LumiTracker Mito Orange (1:1000, 2252, Lumiprobe, Moscow, Russia) for mitochondrial membrane potential, and 6-Carboxy-H_2_DCFDA (1:1000, 3290, Lumiprobe, Moscow, Russia) for reactive oxygen species (ROS) detection. Imaging was performed immediately without fixation.

### 2.7. Image Acquisition and Analysis

Confocal imaging was performed using Zeiss LSM 880 Airyscan (Carl Zeiss AG, Oberkochen, Germany), Leica TCS SP5 (Leica Microsystems, Wetzlar, Germany), Leica Thunder Imager (Leica Microsystems, Wetzlar, Germany), or Olympus Fluoview FV10i (Olympus Corp., Tokyo, Japan) microscopes. To ensure quantitative comparability, all acquisition parameters (laser power, gain, pinhole, detector offset) were standardized and kept constant between control and experimental groups within each replicate. Care was taken to avoid pixel saturation in high-intensity regions during acquisition. Image analysis was conducted using FIJI/ImageJ (version 2.9.0), open source, available at https://imagej.net/software/fiji/ (URL accessed on 9 March 2026).

Quantification of immunoreactivity was performed by measuring the mean gray value intensity in specific regions of interest (ROI). The ROI selection strategy was tailored to the embryonic stage and experimental design. For general blastocyst analysis, three independent cytoplasmic regions (excluding the nuclear region) in both the trophectoderm (TE) and inner cell mass (ICM) cells were selected as ROIs, and their values were averaged. In experiments utilizing two-cell embryos, three independent cytoplasmic regions (excluding the nuclear region) were similarly selected and averaged. For the cystamine experiments, where subcellular compartmentalization was critical, three independent cytoplasmic regions and three independent nuclear regions were selected for each blastocyst, analyzed separately in the TE and ICM cells, and averaged. To account for non-specific noise, a background ROI was measured in a cell-free area outside the embryos, and this background signal was subtracted from the embryonic ROI values before quantitative fluorescence calculation. To ensure appropriate normalization across independent experimental batches, the background-subtracted mean gray values were normalized to the mean of the corresponding control group within each biological replicate. Image analysis was performed by an investigator blinded to the experimental group allocation to prevent observer bias—file names were coded prior to analysis.

### 2.8. Statistical Analysis

To rigidly prevent pseudoreplication, individual embryos were not treated as independent statistical units. In all cases, *n* refers to the number of independent biological replicates (distinct litters, dams, or embryo pools) to minimize litter-specific effects. Specifically, for in vivo experiments, *n* reflects the number of independent litters (dams) analyzed. For in vitro experiments, assays were performed on randomized oocytes/embryos derived from at least 3–4 distinct litters, where *n* represents the number of experimental replicates (source litters).

No statistical methods were used to predetermine sample sizes; rather, they were established based on previous experience and comparable studies in the field to ensure sufficient statistical power. Data were analyzed using GraphPad Prism 8.0.1 (GraphPad Software, San Diego, CA, USA). The normality of data distribution was explicitly evaluated using the Shapiro–Wilk test. Based on the distribution and experimental design, differences between two groups were analyzed utilizing the Mann–Whitney U test for independent embryo pools obtained in in vivo experiments, or the Wilcoxon matched-pairs signed rank test for in vitro experiments where embryos from each single litter were equally divided across experimental groups. Multiple group comparisons were conducted using the Friedman test followed by Dunn’s multiple comparisons test. Data are presented as mean ± standard error of the mean (SEM) to visually communicate the magnitude of pharmacological effects and facilitate direct comparison with prior literature, while all inferential statistical conclusions are drawn strictly from the rank-based nonparametric tests. A *p*-value < 0.05 was considered statistically significant.

## 3. Results

### 3.1. Preimplantation Embryos Possess a Dynamic Serotonin Regulation System Mediated by MAO-A

**Figure 1:**
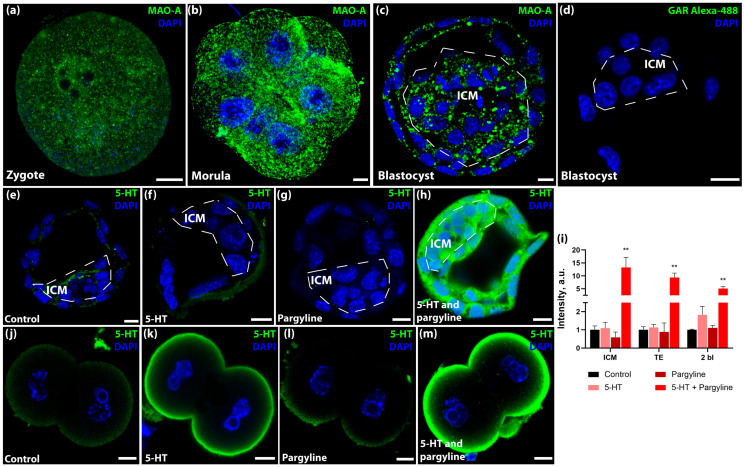
Regulation of intracellular serotonin (5-HT) levels during preimplantation development via monoamine oxidase (MAO) activity. (**a**–**c**) Representative confocal images showing immunolocalization of MAO-A in a zygote (**a**), non-compact morula (**b**), and blastocyst (**c**). Note the punctate cytoplasmic distribution and dense clusters in the interblastomere zones, inner cell mass (ICM), and trophectoderm (TE). (**d**) Negative control (secondary antibodies only). (**e**–**h**) 5-HT turnover in blastocysts incubated in control KSOM medium (**e**), 1 μM 5-HT (**f**), 5 μM pargyline (MAO inhibitor) (**g**), or 5-HT + pargyline (**h**). (**i**) Quantification of 5-HT fluorescence intensity in blastocysts and 2-cell embryos (*n* = 13 independent litters each). Data are presented as mean ± SEM. Asterisks indicate significant differences compared to the control group (Friedman test with Dunn’s multiple comparisons test: ** *p* < 0.01). (**j**–**m**) 5-HT turnover in 2-cell embryos treated as in panels (**e**–**h**). Scale bars: 10 μm.

To determine whether early embryos possess the machinery to regulate intracellular 5-HT levels, we first analyzed the expression and localization of MAO-A, the key enzyme responsible for 5-HT degradation. Immunostaining revealed the presence of MAO-A protein throughout preimplantation development, from the zygote to the blastocyst stage (Figure 1a–c). In zygotes and cleavage-stage embryos, MAO-A exhibited a punctate distribution pattern throughout the cytoplasm, consistent with mitochondrial localization. By the morula stage, we observed the formation of dense MAO-A clusters, particularly in the interblastomere zones (Figure 1b). In blastocysts, the enzyme was ubiquitously expressed in both the inner cell mass (ICM) and the trophectoderm (TE) (Figure 1c). Notably, semi-quantitative analysis indicated a trend toward increased MAO-A immunoreactivity during the transition from zygote to blastocyst, suggesting a developmental upregulation of the degradation machinery.

To assess the functional activity of this system, we performed a serotonin challenge assay. Incubation of blastocysts with exogenous 5-HT (1 μM) alone did not result in intracellular 5-HT accumulation, indicating efficient degradation or efflux. However, co-incubation with the MAO inhibitor pargyline (5 μM) led to a dramatic increase in intracellular 5-HT signal in both TE and ICM cells (Figure 1e–i; *p* < 0.0001). This confirms that blastocysts possess active serotonin active uptake mechanisms balanced by potent MAO-A-mediated degradation. A similar, though less pronounced, effect was observed at the 2-cell stage (Figure 1i–m), indicating that the serotonin turnover system is functional from the onset of development but becomes more robust by the blastocyst stage.

**Figure 2:**
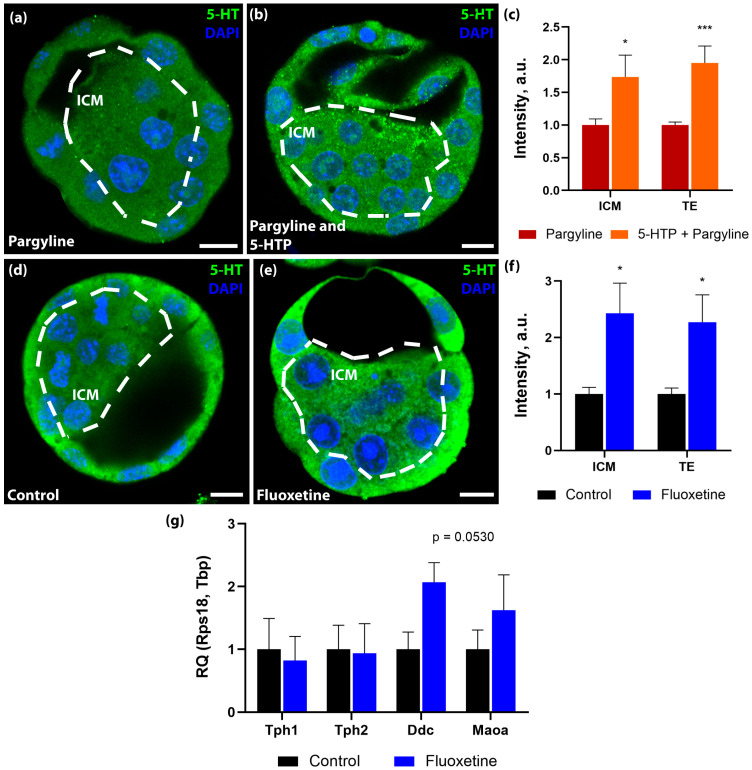
Embryonic capacity for endogenous 5-HT synthesis and compensatory response to altered maternal systemic 5-HT. (**a**,**b**) *De Novo* synthesis assay. Representative images of blastocysts incubated with pargyline alone (**a**) or pargyline + 5-hydroxytryptophan (5-HTP) (**b**). (**c**) Quantification of 5-HT intensity in the TE and ICM (*n* = 11 litters). (Wilcoxon matched-pairs signed rank test: * *p* < 0.05, *** *p* < 0.001). (**d**,**e**) Representative images of 5-HT in blastocysts from control females (**d**) and females treated with oral fluoxetine (**e**). (**f**) Quantification showing compensatory 5-HT accumulation in fluoxetine-exposed embryos (Control *n* = 6 litters, Fluoxetine *n* = 5 litters; Mann–Whitney U test: * *p* < 0.05). (**g**) RT-qPCR analysis of 5-HT synthesis (*Tph1*, *Tph2*, *Ddc*) and degradation (*Maoa*) genes following fluoxetine exposure. Relative quantity is normalized to *Tbp* and *Rps18* (*n* = 7 independent embryo pools). No significant differences were observed between groups, although *Ddc* showed a near-significant trend (*p* = 0.0530; Mann–Whitney U test). Data are presented as mean ± SEM. Scale bars: 10 μm.

Furthermore, we investigated the embryo’s capacity for autonomous serotonin synthesis. Incubation of zygotes with the serotonin precursor 5-HTP in the presence of pargyline resulted in significant 5-HT accumulation compared to pargyline alone (Figure 2a–c). This demonstrates that the enzymatic machinery for serotonin synthesis by Aromatic L-amino acid decarboxylase (DDC) is catalytically active prior to implantation.

### 3.2. Maternal Serotonin Transporter Blockade Triggers Compensatory Endogenous Synthesis in the Embryo

Given the embryo’s theoretical capacity for synthesis, we investigated its response to altered maternal systemic serotonin. We utilized an in vivo model wherein pregnant dams were treated with fluoxetine for 10 days. We hypothesized that blocking maternal SERT would deplete distinct embryonic serotonin pools. Contrary to expectations, immunostaining of 3.5 dpc blastocysts recovered from fluoxetine-treated females revealed a significant increase in intracellular 5-HT levels compared to controls (43% increase in ICM and 43% in TE; Figure 2d–f).

To elucidate the mechanism behind this paradoxical accumulation, we analyzed the expression of genes encoding serotonin synthesis enzymes (*Tph1*, *Tph2*, *Ddc*) and the degradation enzyme (*Maoa*) using RT-qPCR. Overall statistical analysis revealed no formally significant differences in the mRNA levels of these targets between the groups (*Tph1*: *p* = 0.7104, FC = 0.82; *Tph2*: *p* > 0.9999, FC = 0.94; *Maoa*: *p* = 0.5350, FC = 1.62). However, we observed a moderate, non-significant trend toward the upregulation of *Ddc* expression in embryos exposed to maternal fluoxetine (*p* = 0.0530, FC = 2.07, Figure 2g). While a robust transcriptional activation of the entire synthetic pathway was not detected, these data hint at a possible compensatory response, though the lack of statistical significance precludes definitive conclusions. It is plausible that the preimplantation embryo senses the blockade of exogenous serotonin transport and subtly adjust its endogenous biosynthetic cascade—particularly at the final decarboxylation step mediated by DDC—in an attempt to maintain or overshoot homeostatic 5-HT levels.

### 3.3. Intracellular Serotonin Accumulation Does Not Compromise Developmental Potential or Mitochondrial Health

**Figure 3:**
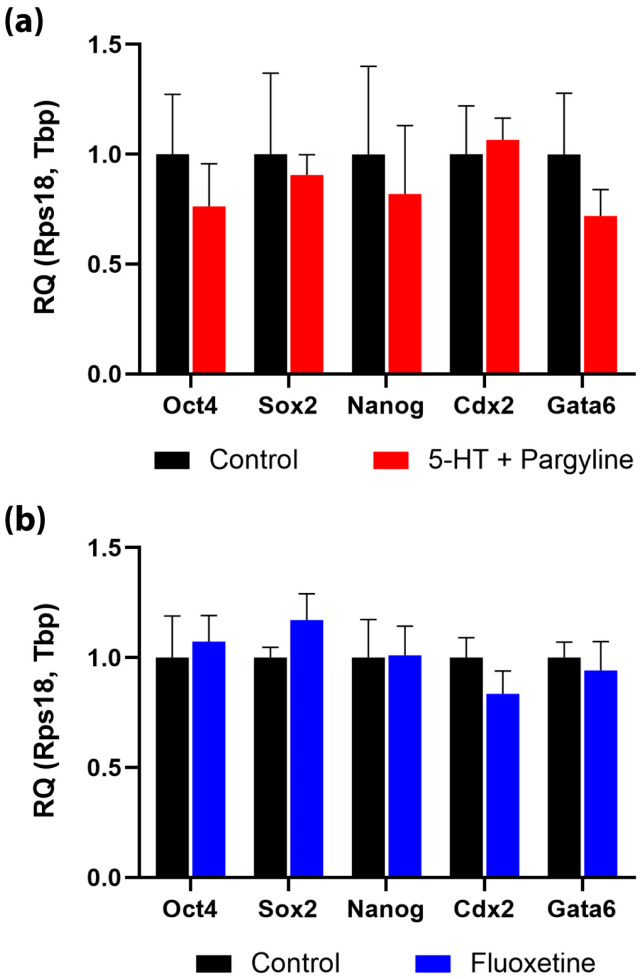
Pluripotency and lineage specification markers remain stable under 5-HT load. RT-qPCR analysis of pluripotency (*Oct4*, *Sox2*, *Nanog*) and lineage specification (*Cdx2*, *Gata6*) markers in blastocysts following (**a**) in vitro 5-HT loading (Pargyline + 5-HT, *n* = 4 embryo pools) and (**b**) in vivo maternal fluoxetine exposure (*n* = 7 embryo pools). Relative quantities were calculated using the 2^−ΔΔCt^ method normalized to *Tbp* and *Rps18*. Data are presented as mean ± SEM. No significant differences were observed between experimental and control groups (Mann–Whitney U test).

We next asked whether the observed hyper-accumulation of serotonin—either induced pharmacologically (in vitro pargyline+5-HT) or via maternal SSRI exposure (in vivo fluoxetine)—exerts toxic effects on embryo development. Analysis of cell fate markers by qPCR revealed that neither treatment regime altered the expression levels of pluripotency genes (*Oct4*, *Sox2*, *Nanog*) or lineage-specification markers (*Cdx2* for trophectoderm, *Gata6* for primitive endoderm) (Figure 3a,b).

**Figure 4:**
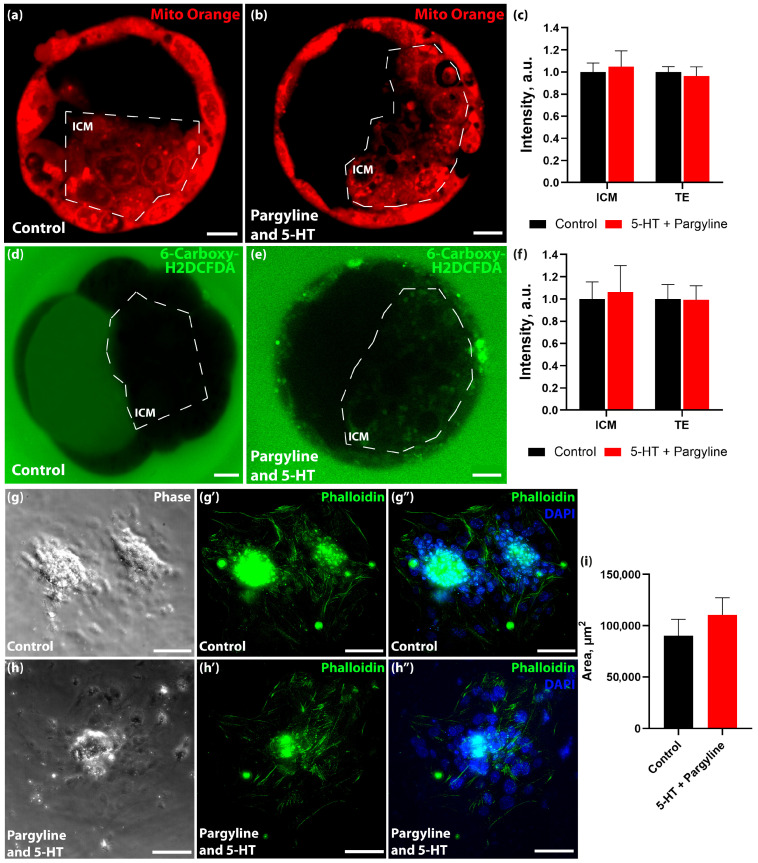
5-HT accumulation does not compromise developmental competence, mitochondrial function, or redox status. (**a**,**b**) Mitochondrial membrane potential (Mito Orange dye) in control (**a**) and 5-HT-loaded (**b**) blastocysts. (**c**) Quantification of Mito Orange intensity (*n* = 13 litters). (**d**,**e**) Oxidative stress evaluation (ROS sensor 6-Carboxy-H2DCFDA) in control (**d**) and 5-HT-loaded (**e**) blastocysts. (**f**) Quantification of ROS levels (*n* = 15 litters). (**g**,**h**) In vitro implantation assay. Representative blastocyst outgrowths at 8.5 dpc cultured in standard medium (**g**) or pre-incubated with pargyline + 5-HT (**h**). (**i**) Quantification of blastocyst outgrowth area (*n* = 13 litters). No significant differences were detected in any of the assessed parameters across all panels (Mann–Whitney U test). Data are presented as mean ± SEM. ICM: inner cell mass, TE: trophectoderm. Scale bars: 10 μm for (**a**,**b**) and (**d**,**e**), 100 μm for (**g**,**h**).

To assess physiological fitness, we evaluated mitochondrial function and oxidative stress levels in 5-HT-loaded blastocysts. Staining with voltage-dependent Mito Orange dye and the ROS sensor 6-Carboxy-H_2_DCFDA showed no significant differences in mitochondrial membrane potential or reactive oxygen species levels between control and 5-HT-loaded groups (Figure 4a–f).

Finally, we assessed developmental competence using an in vitro implantation assay. Blastocysts loaded with serotonin attached to the substrate and formed characteristic outgrowths with the same efficiency and morphology as controls over an observation period up to 8.5 dpc (Figure 4g–h’’). Collectively, these results demonstrate that the blastocyst is phenotypically robust to fluctuations in intracellular serotonin levels.

### 3.4. Serotonin Overload Drives Transglutaminase-Dependent Histone H3 Serotonylation in Both Nuclear and Cytoplasmic Compartments

**Figure 5:**
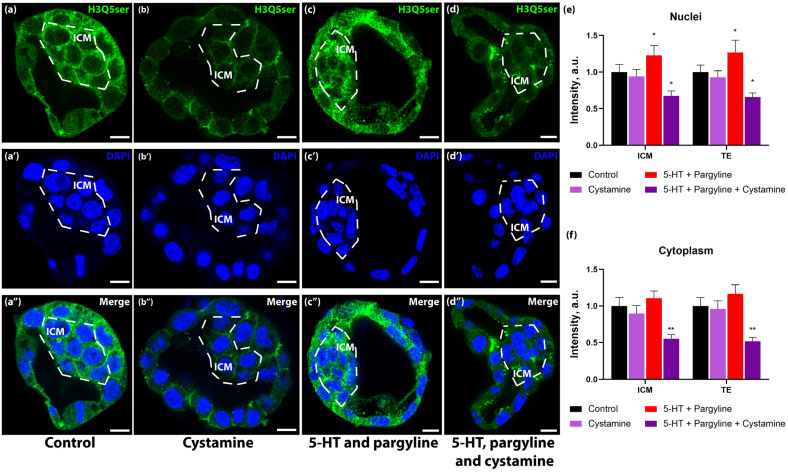
5-HT load drives transglutaminase-dependent accumulation of H3Q5ser in nuclear and cytoplasmic compartments. (**a**–**d**) Representative confocal images of blastocysts immunostained for serotonylated histone H3 (H3Q5ser) cultured in: (**a**) Control KSOM; (**b**) Cystamine (broad-spectrum transglutaminase inhibitor); (**c**) Pargyline + 5-HT; and (**d**) Pargyline + 5-HT + Cystamine. (**e**,**f**) Quantification of H3Q5ser immunoreactivity in nuclei (**e**) and cytoplasm (**f**). 5-HT loading significantly increases H3Q5ser levels, which is reversed by cystamine (*n* = 18 litters). Data are presented as mean ± SEM. Asterisks indicate significant differences compared to the control group (Friedman test with Dunn’s multiple comparisons test: * *p* < 0.05, ** *p* < 0.01). Scale bar: 10 μm.

Since serotonin accumulation did not manifest in immediate transcriptional or morphological defects, we investigated whether the excess monoamine was diverted into post-translational modifications, specifically serotonylation. Using antibodies specific for serotonylated histone H3 (H3Q5ser), we detected a basal signal in control blastocysts. However, loading blastocysts with serotonin led to a marked increase in H3Q5ser immunoreactivity (Figure 5b,d). Quantification revealed that this increase occurred not only in the nuclei (+19.6% in ICM, +26.6% in TE) but also prominently in the cytoplasm (+14.3% in ICM, +18.1% in TE). While the nuclear signal corresponds to canonical chromatin modifications, the cytoplasmic signal likely represents the serotonylation of the soluble histone pool known to exist in rapidly dividing early embryos, or potentially other cytosolic proteins targeted by transglutaminases. To confirm the enzymatic nature of this modification, we treated embryos with cystamine, a broad-spectrum transglutaminase inhibitor. Cystamine treatment effectively abolished the serotonin-induced increase in H3Q5ser signal, reducing it to levels below baseline (Figure 5c,d). This indicates that excess intracellular serotonin is actively conjugated to histone H3 (and potentially the soluble cytoplasmic histone pool) via a transglutaminase-dependent mechanism, serving as a biochemical “record” of serotonin exposure. Interestingly, co-treatment with cystamine not only prevented the induced increase but reduced basal H3Q5ser levels below control values (Figure 5d,f). This suggests that high intracellular serotonin flux may trigger rapid turnover of serotonylated proteins, leading to signal depletion when the restoring TG2 is inhibited.

## 4. Discussion

In this study, we demonstrate that the preimplantation mouse embryo is not merely a passive recipient of maternal neuroactive substances but acts as an autonomous regulator of its own serotonergic homeostasis. We identified a multi-layered protective machinery—comprising degradation, transport control, and compensatory synthesis—that maintains embryonic fitness. Furthermore, we provide evidence for a non-canonical signaling pathway wherein excess serotonin has the potential to act as an epigenetic modifier via transglutaminase-mediated serotonylation of histone H3.

We observed that MAO-A expression begins as early as the zygote stage and progressively intensifies towards the blastocyst stage. This aligns with recent evidence suggesting that components of the serotonergic system are functional well before implantation [41,42,43]. The punctate localization of MAO-A, consistent with mitochondrial association, suggests it functions as a “metabolic firewall,” protecting the embryo’s energy generator from monoamine toxicity [44,45]. Interestingly, our turnover assays revealed a temporal lag: while the enzyme is physically present in the zygote, its full functional capacity to degrade massive serotonin loads matures by the blastocyst stage. We must note a pharmacological limitation regarding these functional turnover assays: the inhibitor utilized, pargyline, is not strictly isoform-specific and blocks both MAO-A and MAO-B. While our localization studies specifically confirm the robust expression of MAO-A in these embryos, we cannot completely rule out a complementary functional contribution from MAO-B during the pharmacological blockade. This delay may reflect the gradual activation of the zygotic genome [46] or a biological threshold requirement wherein the enzyme activates only when serotonin concentrations exceed a critical level [47]. The robustness of this system at the blastocyst stage is physiologically coherent, as this period coincides with preparation for implantation—a window where MAO-A activity is known to be critical. Low MAO-A activity has been linked to implantation failure [48], while adequate expression supports successful attachment [29,49].

A central finding of our work is the demonstration of the embryo’s biochemical capacity for *De Novo* serotonin synthesis. While historical dogma viewed embryonic serotonin as exclusively maternal in origin [50,51], our data show that when provided with the precursor (5-HTP), the preimplantation embryo possesses the functional enzymatic machinery to synthesize 5-HT independently. However, we must strictly distinguish between this biochemical capability and baseline physiological function. Our precursor-loading experiments functionally validate the enzymatic capacity, but they do not necessarily imply that the embryo continuously relies on autonomous serotonin production under unperturbed physiological conditions in vivo. This latent autonomy is most strikingly illustrated by the “fluoxetine paradox.” We anticipated that maternal treatment with an SSRI would deplete embryonic serotonin. Instead, we observed a significant hyper-accumulation of 5-HT in blastocysts, accompanied by a trend suggestive of a compensatory upregulation of the biosynthetic gene Ddc, although this shift did not reach formal statistical significance. This suggests a sophisticated feedback loop: the embryo potentially senses the blockade of exogenous transport (SERT) and metabolically reprograms itself to activate this latent synthetic capacity and produce its own serotonin. A critical question arises: how does the embryo fuel this synthesis if SERT is blocked? While fluoxetine inhibits the transport of serotonin itself, it does not block the transport of amino acid precursors. As a working hypothesis, we propose that the embryo might utilize the L-type amino acid transporter (LAT1/SLC7A5), known to be expressed in blastocysts [52], to uptake maternal tryptophan or 5-HTP from the oviductal fluid, thereby bypassing the SERT blockade to fuel DDC-mediated synthesis. However, we acknowledge that this compensatory pathway currently remains inferential without direct measurements of substrate availability. Future studies utilizing targeted metabolomics or tracing of labeled precursors are required to definitively validate intracellular transport kinetics under these conditions. Nevertheless, these findings have profound implications for reproductive toxicology. They imply that SSRIs do not simply “starve” the embryo of serotonin but induce a state of hyper-synthesis and metabolic shift. Unlike post-implantation stages, where SSRIs are often linked to malformations [53], the preimplantation blastocyst appears capable of robust molecular compensation. Nevertheless, we acknowledge a limitation regarding our in vivo pharmacological model: the lack of direct analytical quantification of fluoxetine and its active metabolite (norfluoxetine) in the maternal circulation or reproductive tract for this specific cohort limits our ability to make definitive claims regarding causative local drug concentrations and the observed embryonic phenotype.

Despite the dramatic biochemical shifts induced by MAO blockade or fluoxetine exposure, the embryos remained phenotypically normal. It has been previously reported that serotonin signaling can suppress the induction of pluripotency, favoring the maintenance of a differentiated cellular status [54]. However, in our model, we detected no impairment in morphology, implantation competence, mitochondrial potential, or ROS levels. Furthermore, the expression of key pluripotency (*Oct4*, *Nanog*) and specification markers (*Cdx2*, *Gata6*) remained stable. This contradicts in vitro models suggesting that serotonin synthesis blocks reprogramming [54] or mesodermal differentiation [36], likely because the intact embryo possesses buffering systems absent in isolated cell cultures.

However, “phenotypically normal” does not mean “unchanged.” If the excess serotonin is not toxic and does not alter gene expression profiles immediately, where does the signal go? Our data suggest it is encoded epigenetically through protein serotonylation. We identified H3Q5ser immunoreactivity as a robust marker of serotonin load. This modification was observed not only in the nucleus but prominently in the cytoplasm. We acknowledge that antibody-based detection in the cytoplasm warrants cautious interpretation, as transglutaminases can serotonylate various cytosolic proteins, including small GTPases and cytoskeletal components [55]. However, significant biochemical evidence points to the existence of a substantial pool of soluble, non-chromatin-bound histones in early embryos, required for rapid cleavage divisions [56]. We propose that the cytoplasmic H3Q5ser signal largely reflects the “pre-loading” or “mark-and-store” of these soluble histones prior to their nuclear import. Importantly, we must explicitly emphasize that we have not directly demonstrated that this cytoplasmic staining exclusively corresponds to altered histones. Because transglutaminases can modify a broad array of targets, the exact molecular identity of the cytoplasmic proteins undergoing serotonylation in our model remains to be conclusively established. Regardless of the specific protein target, the cystamine-sensitive nature of this signal confirms it is a transglutaminase-dependent modification that may serve as a molecular imprint of serotonin stress. However, it is pertinent to acknowledge that cystamine acts as a pan-transglutaminase inhibitor. Although tissue transglutaminase (TGM2) is the enzyme classically responsible for monoaminylation, our current pharmacological evidence does not allow us to pinpoint the precise transglutaminase isoform mediating this specific embryonic response. We acknowledge, however, a broader technical limitation of the current study: the extreme scarcity of biomaterial associated with preimplantation embryos makes orthogonal biochemical validation (such as Western blotting or mass spectrometry of specific epigenetic marks) practically unfeasible. Consequently, our findings rely strictly on immunofluorescence supported by targeted pharmacological inhibition. Definitive proteomic mapping of this modification and the exact downstream targets of cytoplasmic serotonylation will require future advances in ultra-low input mass spectrometry. This mechanism aligns with the concept of “permissive” chromatin modifications described in neurons, where H3Q5ser promotes TFIID binding and gene activation [57]. In the context of the embryo, we speculate that this could serve as a mechanism potentially linked to the Developmental Origins of Health and Disease (DOHaD). However, we emphasize that our current experimental endpoints are limited to preimplantation phenomena, namely morphology, implantation-like outgrowth, mitochondrial potential, ROS levels, and a targeted gene expression panel. Therefore, directly linking these early biochemical alterations to long-term developmental programming remains strictly hypothetical. Future longitudinal studies tracking post-implantation development are necessary to determine if the embryo survives the serotonin stress without immediate morphological defects (phenotypic buffering), but its chromatin landscape is chemically modified. While we did not observe immediate changes in pluripotency markers, such modifications (H3Q5ser) are known to exert latent effects that manifest later during differentiation [58,59].

Our findings underscore the critical importance of maintaining a precise serotonin concentration balance in the microenvironment of the preimplantation mammal. The equilibrium between extracellular and intracellular serotonin is a fundamental factor influencing long-term developmental outcomes across diverse animal phyla [60,61]. In mammals, deviations in either direction carry risks. On one hand, the depletion of intracellular serotonin—observed, for instance, during SERT blockade in oocytes—negatively impacts reproductive output and maturation competence [20]. On the other hand, states of elevated serotonin can induce latent consequences via the epigenetic mechanisms described here. Such serotonin overload is not merely an experimental construct but a clinical reality associated with ovarian hyperstimulation syndrome (OHSS) [62], inflammatory processes, and other pathological conditions [63,64]. Therefore, maintaining stable serotonin homeostasis in the ovary and reproductive tract is essential. During follicular development, the oocyte is shielded by a barrier of granulosa cells that actively uptake and degrade serotonin [26]. However, our data suggest that following ovulation, the embryo must activate its own autonomous mechanisms to control serotonin exposure, effectively taking the baton from the follicle to ensure developmental success.

## 5. Conclusions

We propose that the preimplantation embryo acts as a “fortress” with a semi-permeable gate. It utilizes MAO to degrade excess maternal serotonin but switches to endogenous synthesis if the supply is cut off. Crucially, when serotonin levels fluctuate, the signal is not lost but is chemically “written” onto histone H3 via transglutaminase activity. We hypothesize that this serotonylation could potentially represent a molecular scar—a theoretical mechanism by which early environmental exposures (such as maternal antidepressant use) are recorded in the epigenome without disrupting immediate survival. However, extended in vivo studies tracking embryos beyond the preimplantation stage are essential to validate whether these changes truly impact long-term developmental programming.

## Figures and Tables

**Table 1:** Primer sequences used for qPCR.

Gene Name	NCBI Gene ID	Forward Primer	Reverse Primer
*Cdx2*	12591	AGTCCCTAGGAAGCCAAGTGAA	TCTCGGAGAGCCCAAGTGT
*Ddc*	13195	AGCGGGAAGCCTTTATCTCTGTCT	CCTCCGGGCCTGTGTAGTGTC
*Gata6*	14465	ATGCATGCGGTCTCTACAGC	CCCTCAGCATTTCTACGCCA
*Maoa*	17161	GCTGAGGAATGGGACAAGATAACC	TACCTCCACACTGCCTCACATACC
*Nanog*	71950	CAGATGCAAGAACTCTCCTCCA	CAGATGCGTTCACCAGATAGC
*Oct4*	18999	TGGAGGAAGCCGACAACAAT	AACCATACTCGAACCACATCCTT
*Rps18*	20084	AAGAAAATTCGAGCCCATAGAGG	TAACAGCAAAGGCCCAGAGACT
*Sox2*	20674	TTTGTCCGAGACCGAGAAGC	CTCCGGGAAGCGTGTACTTA
*Tbp*	21374	GTAGCGGTGGCGGGTATCT	CGTCTTCAATGTTCTGGGTTATCT
*Tph1*	21990	TGCGACATCAGCCGAGAACAGT	GGCGCAGAAGTCCAGGTCAGA
*Tph2*	216343	CATGGCTCCGACCCCCTCTACA	ATACGCCCGCAGTTGACCCTCTT

## Data Availability

The original contributions presented in this study are included in the article. Further inquiries can be directed to the corresponding author.

## Abbreviations

The following abbreviations are used in this manuscript:

5-HT	5-hydroxytryptamine (serotonin)
5-HTP	5-hydroxy-L-tryptophan
DDC	Aromatic L-amino acid decarboxylase
DOHaD	Developmental Origins of Health and Disease
dpc	Days post coitum
H3Q5ser	Histone H3 serotonylated at glutamine 5
ICM	Inner cell mass
MAO	Monoamine oxidase
MAO-A	Monoamine oxidase A
OHSS	Ovarian hyperstimulation syndrome
ROS	Reactive oxygen species
RT-qPCR	Reverse Transcription Quantitative Polymerase Chain Reaction
SEM	Standard Error of the Mean
SERT	Serotonin transporter
SSRI	Selective serotonin reuptake inhibitor
TE	Trophectoderm
